# Evaluation of exotic oat (*Avena sativa L*.) varieties for forage and grain yield in response to different levels of nitrogen and phosphorous

**DOI:** 10.7717/peerj.12112

**Published:** 2021-09-23

**Authors:** Hamida Bibi, Suleman Hameed, Mudassar Iqbal, Amal Al-Barty, Hadeer Darwish, Amanullah Khan, Shazma Anwar, Ishaq Ahmad Mian, Murad Ali, Afia Zia, Muhammad Irfan, Maria Mussarat

**Affiliations:** 1 Department of Soil and Environmental Sciences, NWFP Agriculture University, Peshawar, Peshawar, Khyber Pakhtunkhwa, Pakistan; 2 Department of Agricultural Chemistry and Biochemistry, NWFP Agriculture University, Peshawar, Peshawar, Khyber Pakhtunkhwa, Pakistan; 3 Department of Biology, College of Sciences, Taif University, Taif, Saudi Arabia; 4 Department of Biotechnology, College of Sciences, Taif University, Saudi Arabia, Taif, Saudi Arabia; 5 Department of Agronomy, NWFP Agriculture University, Peshawar, Peshawar, khyber Pakhtunkhwa, Pakistan; 6 Cereal Crops Research Institute (CCRI) Pirsabak, Nowshera, Cereal Crops Research Institute (CCRI) Pirsabak, Nowshera, Nowshera, Khyber Pakhtunkhwa, Pakistan; 7 Department of Medicinal and Aromatic Plants, Horticulture Institute, Agriculture Research Center, Egypt

**Keywords:** Oat, Nitrogen, Phosphorous, Yield, *Avena sativa* L., Nutrients

## Abstract

A field experiment was conducted during the Rabi season 2017–2018 (October–March) at the University of Agriculture, Peshawar research farm to examine the influence of different nitrogen (N) and phosphorus (P) levels on two different oat varieties: Australian and Ukrainian. The treatments included control and three levels of nitrogen and phosphorus at 30, 60, and 90 kg ha^−1^. The treatments were arranged in randomized complete block design (RCBD) and replicated three times. The findings showed that the oat varieties were significantly different from one another in yield and yield parameters. The Australian variety recorded higher emergence (49 plants m^−2^), days to emergence (15 days), days to flowering (122 days), days to maturity (145 days), plant height (142.7 cm), number of leaves (6.03 leaves plant^−1^), number of tillers (92.2 tillers m^−1^), biological yield (8,179.2 kg ha^−1^), and grain yield (3,725.6 kg ha^−1^) than the Ukrainian variety. Similarly, different N and P levels, the maximum days to emergence, days to flowering, and days to maturity were recorded in a control plot. The application of 105 kg N + 90 kg P ha^−1^ was statistically similar to the application of 105 kg N + 60 kg P ha^−1^. Maximum emergence (60 plants m^−2^), number of leaves (7.0 leaves plant^−1^), plant height (118.6 cm), number of tillers m^−1^ (102.6), biological yield (9,687.5 kg ha^−1^), and grain yield (4,416.7 kg ha^−1^) were determined in Australian variety. Based on the findings of this study, the Australian variety performed better in terms of yield and yield components and the application of N and P fertilizers at the rate of 105 kg N + 60 kg P ha^−1^ produced the best results in both oat varieties.

## Introduction

Oat (*Avena sativa* L.) belongs to the family Poaceae and ranks as the fourth most important cereal crop worldwide. The oat plant can produce four to five culms with diameters of 0.32 to 0.625 cm and heights ranging from 60 to 150 cm (Gifari, 2020). Oats contain 9.23% fat, 3.56% protein, 30.44% fiber, 0.82% calcium, and 0.27% phosphorous. Its leaves and grains are a rich source of carbohydrates and carotene. Oats can be used like other small grains as a forage crop because it has a finer stem, higher palatability, and fast-growing properties; if harvested at the boot stage, it becomes a good source of silage crop. In Pakistan livestock is a basic component of the agricultural sector, relied on by 30–35% of the rural population. Fodder production is a major limiting factor for livestock production. Demand for fodder exceeds the amount cultivated on 2.0 million hectares (Hussain, 2017). Good quality forage production in large quantities is the basic requirement for a more efficient and productive livestock industry (Rehman et al., 2017). The oat crop is used as fodder for animals and cultivated in different regions of the country due to its diverse adaptability; it can be grown in a wide range of soil types, rainfall situations, and altitudes. However, moderate and cool subtropical situations are ideal for its development.

In Pakistan, oats are grown as a forage crop in winter under irrigated and rain-fed conditions. Fodder crop oat is grown in conditions with well-distributed rainfall of 400 mm and a temperature range of 16–32 °C during the 4 months of its growing seasons. Therefore, most of the Pothohar region is considered suitable to cultivate this fodder crop successfully. Oats as a fodder could play a vital role in improving the productivity of the dairy industry, currently emerging in this region, to meet the increasing demand for milk, butter and meat. Improved varieties of oats can produce three-fold green fodder, *i.e*., 60–80 tons per hectare, and feed more animals per unit area than the traditional fodder crops in the region (Chaudhary, Mukhtar & Saleem, 1985). Various oat varieties have already been evaluated for desirable characteristics, like early to late maturity, high yield, more nutritive qualities, high palatability, multi-cut ability, and disease-resistance, for many agro-ecological zones of the country in general (Hussain et al., 2010) and the Pothowar region in particular (Bhatti, Ashiq & Dost, 1992). The beneficial characteristic of oats is their tolerance to cold and drought, which enables them to be used as green fodder for animals during the lean period (December and January). When hay is rare and the animals in this tract feed on protein-deficient dry stalks of maize, sorghum, and millets. Forage oats produced a high yield in response to higher nitrogen fertilizer doses (Moreira, 1989). Similarly, nitrogen fertilization improved nutritive values of oat grains (Givens, Davies & Laverick, 2004).

Chemical fertilizer is a crucial input and has become an integral part of modern technology for crop production to improve soil fertility. One of several reasons for reduced yield is the low fertility of most cultivated soils globally, soils are especially deficient in nitrogen (Jones, 1982). Among the essential nutrients for plant growth, nitrogen plays a dominant role in plant chlorophyll production as a component of enzymes, proteins, nucleic acids, and cell walls (Carlsson & Huss-Danell, 2003). Nitrogen is also a constituent of low molecular weight plant compounds, including nucleotides, amides, and amines. Consequently, sufficient nitrogen is a requirement for achieving good crop yield and is the key input for achieving the highest yield of maize (Abbas et al., 2005). The application of nitrogen significantly increased the grain yield in oat crops (Jackson et al., 1994). Similarly, phosphorus plays a vital role in growth, yield, and water use efficiency in crops (Rifat & Safdar, 2010; National Fertilizer Development Centre, 1997). Compared to nitrogen, relatively little information is available on the effects of phosphorus (P) and potassium (K) on oat crops. However, the importance of early-season phosphorus supply to the formation of grains in field crops is well-documented (Grant et al., 2001).

Nitrogen supplementation increased forage production and improved quality parameters (Eltelib, Hamad & Ali, 2006). Similarly, phosphorous and nitrogen application at the optimum level significantly increased the yield of oat crops (Mohr et al., 2007). The study of phosphorus application on oat seed coating showed increased biomass production and grain size but did not affect grain yield (Peltonen-Sainio, Kontturi & Peltonen, 2006). In P deficient soils higher oat yield was associated with increased P concentration (Bolland & Brennan, 2005). Different soil factors, including soil P concentration, soil temperature, moisture, pH, texture, bulk density, and plant factors, such as root growth, may affect P supply to the plant (Grant et al., 2001) and potential responses to P fertilizers application. Thus, the present study was carried out to investigate the effects of different levels of N and P on different oat varieties and the influence of varying levels of N and P on nutrient uptake by oat varieties.

## Materials and Methods

The experiment was conducted at the research farm of the University of Agriculture, Peshawar from 2017 to 2018 to study the effects of different levels of N and P on the yield and yield components of two different exotic oat varieties (Australian and Ukrainian). The site had a hot, semi-arid climate and mild to cool winter. The experiment was laid out in randomized complete block design (RCBD) and replicated three times. The two oat varieties, Australian and Ukrainian, used in this experiment were provided by the Department of Plant Breeding and Genetics, the University of Agriculture, Peshawar. Three different levels of N (35, 70, and 105 kg ha^−1^) and P (30, 60, and 90 kg ha^−1^) were applied at sowing time; the recommended basal dose of nutrients for oat crops is 75:50:0 (Hussain, Khan & Imran, 2004). The seed was sown at the rate of 70–80 kg ha^−1^. Diammonium Phosphate (DAP) was used as the P source. Nitrogen was applied in the form of urea in two split doses; the first dose was applied at sowing time, while the second dose was included with the second irrigation. The plot was 3 × 2.5 m^2^ and the distance between the rows was 18–25 cm. All agronomic practices (irrigation, hoeing, and weeding) were carried out uniformly for all the treatments.

The texture of the pre-sowing and post-harvest soil samples was determined by the mechanical method of texture determination, using the textural triangle (Gee, 1986). The soil pH was determined by a pH meter (McLean, 1983). The electrical conductivity (EC) of soil indicates the total soluble salts in the soil; thus, EC was determined with a Jenway 4510 EC meter (Richard, 1954). The percentage of organic matter content in pre-sowing and post-harvest soil was calculated with the Nelson & Sommers (1996) method, while the total N content in the soil samples was determined by the Bremner (1982) method. The P and K contents in the samples were determined by the Soltanpour & Schwab (1977) test.

The agronomic data as days to emergence was recorded by counting the number of days from the sowing date to the date that 75% of the seedlings emerged. In contrast, emergence m^−2^ was recorded by counting the total number of plants that emerged in 1 m^−2^ area in a row in each plot. Two random rows were selected in each plot, the number of plants m^−2^ was counted, and the averaged values were recorded as emergence m^−2^. The average number of leaves per plant was counted in ten randomly selected plants from each plot. At the harvesting stage, five randomly selected plants were measured from their tops to the ground using a measuring tape to collect the plant height data (cm). At the same time, days to maturity were counted from the date of sowing to the date on which at least 50% of the plants in each plot reached physiological maturity by visual observation. Data on the days to flowering was recorded from the sowing date to the first flowering of about 50% of the plants in each plot. Tillers m^−1^ were calculated in row length by counting all productive and non-productive tillers in each plot.

The biological yield was obtained by harvesting plants from four central rows of each plot and, after drying and weighing, converting them into kg ha^−1^. Grain yield was recorded after oat plants were harvested from four central rows of each treatment and the yield was converted into kg ha^−1^ by weighing the grains obtained after threshing. The nutritional status of the grains was determined by collecting plant samples, washing them with distilled water, and oven-drying them for 2 days (48 h) at 70 °C. The dried samples were ground and stored in bottles. The total N in the plant samples was determined by the Kjeldahl method (Bremner & Mulvaney, 1982), while the wet digestion method (Richard, 1954) was used to determine P content in the plant samples. The data were statistically analyzed using analysis of variance appropriate for randomized complete block design. The mean results were compared using the least significant difference (LSD) test at a 5% level of significance and whether the F-values were significant was determined (Steel & Torrie, 1980).

## Result and discussion

A field experiment was conducted to study the effect of different levels of nitrogen and phosphorus on yield and yield parameters of two oat varieties at the research farm at the University of Agriculture, Peshawar during the 2017 to 2018 Rabi season. A composite soil sample was taken from the research area and analyzed for various physicochemical properties; the results showed that the research site was silt loam in texture with alkaline pH and non-saline with high bulk density. At the same time, organic matter content and nitrogen concentration were low and available P and K were in the medium range (Table 1).

**Table 1:** Physico-chemical properties of the soil of the experimental site.

Properties	Units	Concentration
Silt	%	65.8
Sand	%	30.3
Clay	%	3.9
Texture Class	–	Silt loam
pH	–	7.613
EC	dS m^−1^	0.173
Bulk Density	g cm^−3^	1.26
Organic Matter	%	0.79
Total Nitrogen	%	0.07
P (AB-DTPA extractable)	mg kg^−1^	3.67
K (AB-DTPA extractable)	mg kg^−1^	81.42

The data on the days to plant emergence was non-significantly influenced the oat varieties; however, different nitrogen and phosphorus levels significantly influenced the emergence rate (Table 2). More days to emergence, 18, were recorded in the control treatment where no nutrients were applied in the form of fertilizers, while fewer days to emergence, 13, were recorded in a treatment where 105 kg N and 90 kg P were applied. The interaction of oat varieties, nitrogen, and phosphorus levels were non-significant. Due to the lack of nutrients, days to emergence was delayed in the control plot. Simultaneously, the availability of a high amount of nitrogen and phosphorus enhanced the emergence of plants m^−2^; similarly, the application of mineral fertilizers in wheat crop showed a significant positive response of emergence m^−2^ (Ferrari et al., 2016). The emergence of plants m^−2^ was non-significantly affected between the oat varieties (Australian and Ukranian) while significantly affected by different nitrogen and phosphorus levels (Table 3). The availability of nutrients increased the emergence number m^−2^. The treatment receiving 105 kg N and 90 kg P resulted in the maximum emergence m^−2^, while the control plot showed significantly less emergence m^−2^. A lack of sufficient nutrients reduced the emergence rate in the control plot. The significant influence of nutrient reserves on seed emergence has also been reported in other studies (Kołodziejek, 2017).

**Table 2:** Days to emergence as affected by the application of different levels of N and P to the oat varieties where NS stand for non-significant.

Treatments		Australian	Ukrainian	Mean
kg ha^−1^				
N	P			
0	0	18	19	18a
35	30	16	15	16b
35	60	15	15	15b
35	90	16	15	15b
70	30	16	15	16b
70	60	15	14	15b
70	90	14	15	14bc
105	30	15	15	15bc
105	60	13	13	13c
105	90	13	13	13c
Mean		15a	15a	

**Note:**

LSD for varieties = NS, LSD for fertilizers = 1.75 * LSD for V × F = NS.

Letter ‘a’ represent maximum value while ‘c’ represent minimum value.

**Table 3:** Emergence m^2^ as affected by the application of different levels of N and P to the oat varieties.

Treatments		Australian	Ukrainian	Mean
kg ha^−1^				
N	P			
0	0	23	31	27f
35	30	46	45	45e
35	60	46	50	48e
35	90	45	46	45de
70	30	49	49	49cde
70	60	54	52	53bcd
70	90	56	55	55abc
105	30	54	54	54ab
105	60	59	57	58ab
105	90	60	61	60a
Mean		49b	50a	

**Note:**

LSD for varieties = NS, LSD for fertilizers = 5.5 * LSD for V × F = NS.

Letter ‘a’ minimum value while ‘f’ represents represents minimun value.

The days to flowering differed by both the varieties and N and P levels (Table 4). Higher days to flowering were recorded in the Australian variety, while the Ukrainian variety flowers took fewer days to emerge. Similarly, maximum days to flowering were observed in the control plot; whereas minimum days to flowering were recorded in the plot where 105 kg N and 90 kg P was applied, these results were statistically similar with the results of treatment where 105 kg N and 60 kg P was applied. The maximum days to flowering in the control plot was due to the limited availability of nutrients as both varieties showed maximum days to flowering in this plot as compared to the other treatments. The significant response of flowering days to the application of nutrients has been found in tomato crops and the significant effect of inorganic fertilizers on days to flowering in oat crops has also been reported (Waheed et al., 2012; Ali et al., 2013).

**Table 4:** Days to flowering as affected by the application of different levels of N and P to oat varieties.

Treatments		Australian	Ukrainian	Mean
kg ha^−1^				
N	P			
0	0	130	110	120a
35	30	127	109	118b
35	60	125	108	117b
35	90	122	106	114c
70	30	123	105	114c
70	60	121	103	112d
70	90	120	102	111d
105	30	117	100	109e
105	60	116	98	107ef
105	90	117	96	107f
Mean		130a	104b	

**Note:**

LSD for varieties = 0.86 * LSD for fertilizers = 1.93 * LSD for V × F = NS.

Letter ‘a’ minimum value while ‘f’ represents represents minimum value.

Statistical analysis of the data showed that the days to maturity were significantly affected by both the varieties and different N and P levels (Table 5). The Australian variety showed more days to maturity due to its genotypic characteristics and late days to flowering. The Ukrainian variety showed fewer days to maturity. Similarly, the control plot showed the maximum days to maturity. In contrast, the minimum days to maturity were calculated with the application of 105 kg N and 90 kg P, which was statistically similar to the treatment where 105 kg N and 60 kg P were applied. The minimum days to maturity in treatment 9 (105 kg N and 90 kg P) were due to the availability of a high amount of major nutrients (Mohr et al., 2007). A positive effect on days to maturity has been observed with applying inorganic fertilizers to oat crops

**Table 5:** Days to maturity as affected by the application of different levels of N and P to oat varieties.

Treatments		Australian	Ukrainian	Mean
kg ha^−1^				
N	P			
0	0	151	131	141a
35	30	149	129	139b
35	60	147	128	137b
35	90	146	127	137c
70	30	146	126	136c
70	60	145	124	134d
70	90	144	123	133e
105	30	143	122	132ef
105	60	140	120	130g
105	90	140	117	129f
Mean		145a	125b	

**Note:**

LSD for varieties = 0.51 * LSD for fertilizers = 1.15 * LSD for V × F = NS.

Letter ‘a’ minimum value while ‘f’ represents represents minimun value.

**Figure 1:**
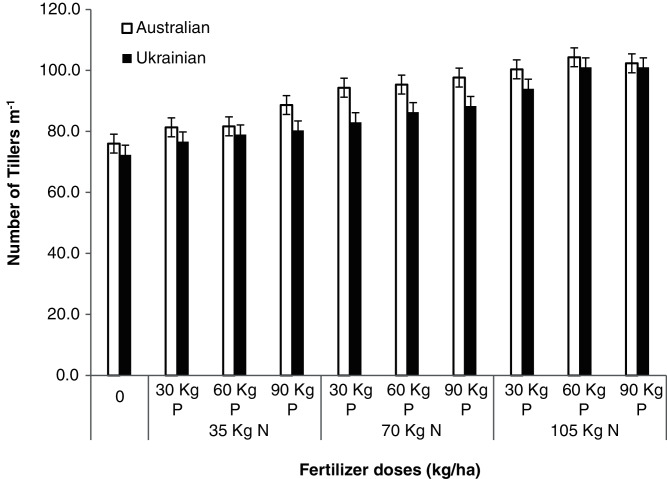
Tillers m^−1^. Tillers m^−1^ of oat varieties as affected by the application of different levels of N and P fertilizers.

The number of tillers m^−1^ was significantly influenced by both the varieties and different N and P levels (Table 6). The Australian variety showed the maximum number of tillers m^−1^while the Ukrainian variety showed a fewer number of tillers m^−1^ because of its genetic influences. The application of different N and P levels influenced the number of tillers m^−1^. The treatment receiving 105 kg N and 60 kg P showed the maximum number of tillers m^−1^; the minimum number of tillers m^−1^ was recorded in a control plot. The increased number of tillers m^−1^ in (105 kg N/ha and 60 kg P/ha) was due to the availability of sufficient soil nutrients. Interaction among oat varieties, N and P levels was significant (Fig. 1). These results are statistically similar to the findings of Waheed et al. (2012) that the application of inorganic fertilizers positively influenced the growth, yield, and quality parameters in oat crops when compared to the control plot.

**Table 6:** Tillers m^−1^ as affected by the application of different levels of N and P to the oat varieties.

Treatments		Australian	Ukrainian	Mean
kg ha^−1^				
N	P			
0	0	76.0	72	74g
35	30	81	77	79f
35	60	82	79	80f
35	90	89	80	85e
70	30	94	83	89d
70	60	95	86	91cd
70	90	98	88	93c
105	30	100	94	97b
105	60	104	101	102a
105	90	102	101	102a
Mean		92a	86b	

**Note:**

LSD for varieties = 1.04 * LSD for fertilizers = 2.33 * LSD for V × F = 3.30*.

Letter ‘a’ minimum value while ‘g’ represents represents minimun value.

Both varieties showed significant changes in the number of leaves per plant as affected by different N and P fertilizers (Table 7). The Australian variety had a increased number of leaves plant^−1^ due to its genetic potential, while a lesser number of leaves plant^−1^ was found in the Ukrainian variety. The application of 105 kg N and 30 kg P showed the maximum number of leaves plant^−1^ due to the availability of sufficient nutrients; the applications of inorganic fertilizers produced a higher number of leaves per plant (Mam Rasul, Ahmed & Ahmed, 2015). In contrast, the minimum number of leaves plant^−1^ was found in the control plot. The significant effect of fertilizer applications on the number of leaves per plant on oat crops has also been reported by Waheed et al. (2012).

**Table 7:** Number of leaves plant^−1^ as affected by the application of different levels of N and P to the oat varieties.

Treatments		Australian	Ukrainian	Mean
kg ha^−1^				
N	P			
0	0	5.11	4.33	4.7f
35	30	5.56	4.67	5.1e
35	60	5.56	5.22	5.4e
35	90	5.54	5.33	5.4e
70	30	6.00	5.67	5.8d
70	60	6.22	5.89	6.1cd
70	90	6.22	6.33	6.3bc
105	30	6.89	6.78	6.8a
105	60	6.89	6.78	6.8a
105	90	6.44	6.56	6.5ab
Mean		6.03a	5.76b	

**Note:**

LSD for varieties = 0.16 * LSD for fertilizers = 0.36 * LSD for V × F = NS.

Letter ‘a’ minimum value while ‘g’ represents represents minimun value.

**Figure 2:**
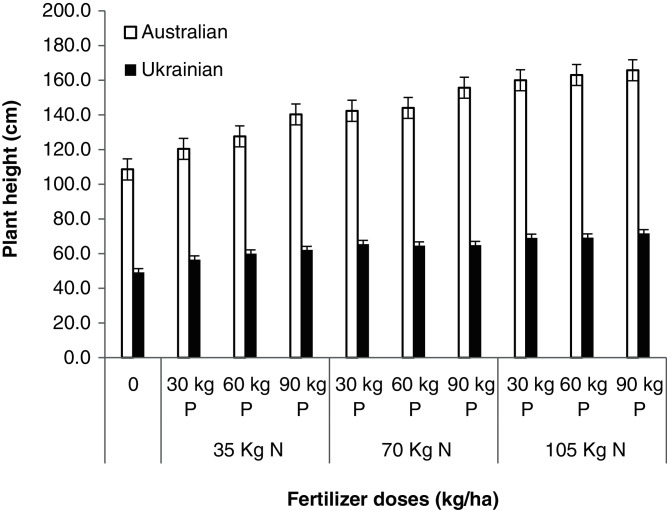
Plant height. Plant height of oat varieties as affected by the application of different levels N and P.

The varieties also showed differences in plant height in different treatments (Table 8). The Australian variety produced taller plants while the Ukrainian variety had shorter plant heights. Similarly, the influence of the application of nutrients revealed that the plant height was taller in the treatments receiving 105 kg N and 90 kg P because the fertilizer applications provided sufficient nutrients to the plants to increase plant height. In comparison, the control plot plants’ height was smaller as shown in Fig. 2. The application of organic and inorganic fertilizers with different ratios increased plant height, spike length, grains per spike, and thousand grains weight in wheat crops (Musaddique et al., 2000). Similar findings were also reported in response to the application of inorganic fertilizers on plant height (Shah et al., 2010). A positive effect of organic and inorganic fertilizers on the height of plants of two different wheat varieties was found by Tripathi, Chander & Meena (2016).

**Table 8:** Plant height (cm) as affected by the application of different levels of N and P to the oat varieties.

Treatments		Australian	Ukrainian	Mean
kg ha^−1^				
N	P			
0	0	108	49	79h
35	30	120	57	88g
35	60	128	60	94f
35	90	140	62	101e
70	30	142	65	104d
70	60	144	65	104d
70	90	156	70	110c
105	30	160	69	115b
105	60	163	69	116b
105	90	165	72	119a
Mean		143a	63b	

**Note:**

LSD for varieties = 0.86 * LSD for fertilizers = 1.92 * LSD for V × F = 2.72*.

Letter ‘a’ minimum value while ‘h’ represents represents minimun value.

The biological yield of the oat crops was positively affected by both the varieties and fertilizer application (Table 9). The Australian variety showed maximum biological yield while minimum biological yield was recorded in the Ukrainian variety. The stem diameters and leaf areas of the Ukrainian variety were larger than the Australian variety due to its genetic makeup. The treatment of 105 kg N and 90 kg P recorded the maximum biological yield while the minimum biological yield was recorded in a control plot. Genetic variations regarding biological yield between different genotypes have been examined by Gevrek & Atasoy (2012). Nitrogen and phosphorous fertilizers significant effect on biological yield has already been reported (Abbas & Fadul, 2013).

**Table 9:** Biological yield (kg ha^−1^) as affected by the application of different levels on N and P to the oat varieties.

Treatments		Australian	Ukrainian	Mean
kg ha^−1^				
N	P			
0	0	6,666.7	6,250.0	6,458.3c
35	30	7,430.6	6,680.6	7,055.6bc
35	60	7,152.8	7,361.1	7,256.9bc
35	90	7,000.0	7,291.7	7,145.8bc
70	30	7,569.4	7,291.7	7,430.6b
70	60	7,569.4	7,291.7	7,430.6b
70	90	8,125.0	7,465.3	7,795.1b
105	30	9,583.3	8,402.8	8,993.1a
105	60	10,277.78	8,333.33	9,305.56a
105	90	10,416.67	8,958.33	9,687.5a
Mean		8,179.2a	7,532.6b	

**Note:**

LSD for varieties = 412.5 * LSD for fertilizers = 922.3 * LSD for V × F = NS.

Letter ‘a’ minimum value while ‘c’ represents represents minimun value.

**Figure 3:**
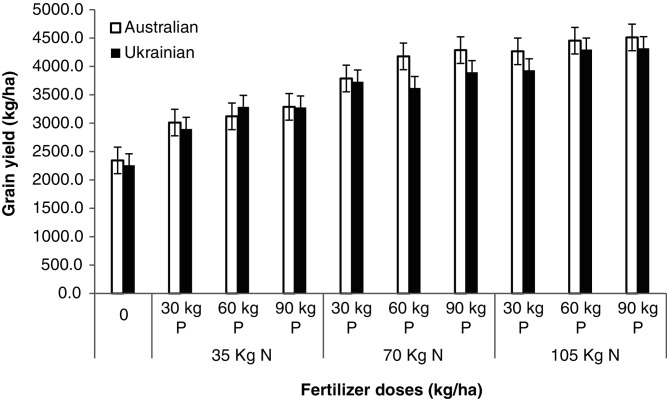
Grains yield. Grain yield of oat varieties as affected by the application of different N and P levels.

Similarly, higher biological yield with increased N and P fertilizer application has also been observed in many studies (Waheed et al., 2012) and the grain yield was significantly affected by both the varieties and different N and P fertilizers (Table 10). The Australian variety produced higher grain yield and had good inherited potential to produce high grain yield, while minimum grain yield was recorded in a plot where the Ukrainian variety was sown. The fertilizer dose influenced the yield as the maximum grain yield was recorded in the treatment receiving 105 kg N and 90 kg P while the minimum grain yield was recorded in a control plot. The interaction among oat varieties, N, and P levels for grain yield was also significant (Fig. 3). The significant differences among oat varieties for grain yield was also reported by Yaseen & Malhi (2009; Gevrek & Atasoy, 2012). Similarly, the significant effect of fertilizer application on the yield of tomato crops has been found by Shah et al. (2010) and Ali et al. (2013). The significant effect of applying significant nutrients on grains yield has also been examined by Mohr et al. (2007).

**Table 10:** Grain yield (kg ha^−1^) as affected by the application of different levels of N and P to the oat varieties.

Treatments		Australian	Ukrainian	Mean
kg ha^−1^				
N	P			
0	0	2,344.4	2,261.1	2,302.8f
35	30	3,011.1	2,900.0	2,955.6e
35	60	3,122.2	3,288.9	3,205.6d
35	90	3,288.9	3,277.8	3,283.3d
70	30	3,788.9	3,733.3	3,761.1c
70	60	4,177.8	3,622.2	3,900.0c
70	90	4,288.9	3,900.0	4,094.4b
105	30	4,266.7	3,933.3	4,100.0b
105	60	4,455.6	4,300.0	4,377.8a
105	90	4,511.1	4,322.2	4,416.7a
Mean		3,725.6a	3,553.9b	

**Note:**

LSD for varieties = 68.468 * LSD FOR fertilizers = 153.10 * LSD FOR V × F = 216.52*.

Letter ‘a’ minimum value while ‘f’ represents represents minimun value.

## Conclusion

Significant differences were observed among the oat varieties for grain yield and yield attributing parameters. The Australian variety had a higher level of emergence m^−2^; days to emergence, maturity, flowering; plant height; number of tillers m^−1^ and leaves plant^−1^; biological yield; and grain yield compared to the Ukrainian variety. Soil parameters as pH, EC, soil total N, and soil P were non-significantly affected with the selected varieties. The application of N and P fertilizers at 105 kg N + 60 kg P ha^−1^ significantly influenced crop yield and yield parameters. Further research on oat varieties in different ecological zones with different sources and nutrient levels is recommended.

## Supplemental Information

10.7717/peerj.12112/supp-1 Supplemental Information 1 Raw data of all parameters.

## Additional Information and Declarations

### Data Availability

The following information was supplied regarding data availability: Raw data are available in a Supplemental File.
